# Supplementary material for: Clinical Utility of Multiplex Ligation-Dependent Probe Amplification in the Genetic Assessment of Patients with Myelodysplastic Syndrome
Source: Biomedicines. 2025 Dec 5;13(12):2985. doi: 10.3390/biomedicines13122985 (PMC12731077; doi:10.3390/biomedicines13122985)
Supplement: Supplementary file 1 [file biomedicines-13-02985-s001.zip › biomedicines-3988668-supplementary.pdf]

Supplementary figure S1a and S1b: Genomic coverage and specifications of the SALSA MLPA probemix P414-C1 MDS.

Length (nt)	SALSA MLPA probe	Chromosomal position (hg18)			Location (hg18) in kb
		Reference	Target	Mutation-specific	
64-105	Control fragments – see table in probemix content section for more information				
118	**ZFY probe** S0135-L13810		Yp11.31		Y-002,889
122	Reference probe 19616-L27299	4p13			04-042,278
127 +	**MIR145 probe** 19041-L29241		5q33.1		05-148,790
133 ~	**IKZF1 probe** 03340-L20689		7p12.2		07-050,338
138	**NCOA2 probe** 09938-L20198		8q13.3		08-071,250
143	Reference probe 14199-L15813	2q13			02-108,894
151	**AATF probe** 21021-L29242		17q12		17-032,463
155	**RPS14 probe** 16292-L18584		5q33.1		05-149,807
160	**MYC probe** 20780-L29239		8q24.21		08-128,822
167	**TP53 probe** 01588-L06028		17p13.1		17-007,531
172	Reference probe 08726-L25038	9q21			09-078,038
176	**SRC probe** 22671-L19475		2q11.23		20-035,448
182	**ETS1 probe** 09496-L25025		11q24.3		11-127,836
186	**KMT2A probe** 19885-L29240		11q23.3		11-117,898
192	**SAMD9L probe** 16293-L24868		7q21.2		07-092,600
197	**KMT2A probe** 17085-L24869		11q23.3		11-117,853
202	**PTK2 probe** 18560-L24870		8q24.3		08-141,739
208 §	**JAK2 probe** 05672-L17742			**p.V617F (c.1849G>T)**	09-005,064
214	**ASXL1 probe** 18515-L25024		2q11.21		20-030,480
220	**SUZ12 probe** 18518-L23809		17q11.2		17-027,345
227	Reference probe 05282-L31924	14q22			14-050,130
232	**SPARC probe** 16294-L24866		5q33.1		05-151,047
238	**EPO probe** 17089-L20201		7q22.1		07-100,158
244	**MET probe** 10323-L10837		7q31.2		07-116,199
250	**MLH1 probe** 18556-L24871		3p22.2		03-037,031
256 ‡	**TP53 probe** 02376-L24176		17p13.1		17-007,519
263	Reference probe 14738-L20398	4q22			04-089,187
268	**MIR146A probe** 15653-L24279		5q33.3		05-159,845
274	**CDKN1B probe** 07949-L24280		12p13.1		12-012,762
282 Ж	**EZH2 probe** 18271-SP0635-L23385		7q36.1		07-148,135
286	**EGR1 probe** 22557-L32018		5q31.2		05-137,829
294	Reference probe 04570-L25187	16q13			16-055,491
301	**EGR1 probe** 17091-L20203		5q31.2		05-137,832
308	**EZH2 probe** 22673-L23019		7q36.1		07-148,145
315	**SPARC probe** 19042-L25092		5q33.1		05-151,027
324	Reference probe 22672-L26274	18q21			18-045,659
332	**TIRAP probe** 18557-L25022		11q24.2		11-125,666
341	**NF1 probe** 02507-L25021		17q11.2		17-026,576

Supplementary fig. S1a

Length (nt)	SALSA MLPA probe	Chromosomal position (hg18)			Location (hg18) in kb
		Reference	Target	Mutation-specific	
348	**RUNX1T1 probe** 09487-L24873		8q21.3		08-093,099
355	**ZMYND8 probe** 14661-L16313		2q13.12		20-045,308
361	**CDK6 probe** 18558-L02523		7q21.2		07-092,085
368	**PRPF31 probe** 06016-L29420		19q13.42		19-059,319
377	Reference probe 10693-L19115	6p12			06-051,721
385 ¬	**FGFR1 probe** 01046-L24278		8p12		08-038,434
392	**ETV6 probe** 13875-L19638		12p13.2		12-011,797
400 ¬	**NIPBL probe** 04837-L24177		5p13.2		05-037,039
409	Reference probe 13405-L31765	6q12			06-065,393
415	**HNF4A probe** 09999-L29451		2q13.12		20-042,477
421	**APC probe** 01807-L29244		5q22.2		05-112,201
427	**KMT2E probe** 18796-L29272		7q22.2		07-104,490
436	Reference probe 08839-L32008	2p13			02-071,767
445	**MECOM probe** 18573-L24179		3q26.2		03-170,332
454	**TP53 probe** 08785-L19640		17p13.1		17-007,515
463	**SMARCA4 probe** 09980-L10439		19p13.2		19-011,000
472	Reference probe 00979-L31258	10p14			10-012,019
481 Ж	**GATA2 probe** 18576-SP0668-L23908		3q21.3		03-129,685
490	**ETV6 probe** 13871-L24874		12p13.2		12-011,914
496	Reference probe 09772-L25949	15q21			15-042,706

Supplementary fig. S1b
Note: The following supporting information can be downloaded at: https://www.mrcholland.com/product/P414?countries=YE#documentation, Product description P414-C1 MDS-v04.pdf, Table 1: SALSA MLPA Probemix P414-C1 MDS [20].

Supplementary Table S1: Detailed MLPA findings in patients with CNVs and point mutations.

Patient №	MLPA Result
1	Normal genotype
2	del 5q22.2; del 5q33.1
3	Normal genotype
4	*JAK2* V617F
5	Normal genotype
6	del 5q22.2; del 5q33.1
7	Normal genotype
8	Normal genotype
9	Normal genotype
10	5q22.2 del; 5q31.2 del; 5q33.1 del
11	Normal genotype
12	Normal genotype
13	5q22.2 del; 5q31.2 del; 5q33.1 del
14	Normal genotype
15	Normal genotype
16	*JAK2* V617F
17	Normal genotype
18	Normal genotype
19	Normal genotype

20	Normal genotype
21	Normal genotype
22	Normal genotype
23	8p12 dup; 8q13.3 dup, 8q21.3 dup; 8q24.21 dup; 8q24.3 dup
24	Normal genotype
25	5q22.2 del; 5q31.2 del; 5q33.1 del
26	20q11.23 del; 20q13.12 del
27	Normal genotype
28	8p12 dup; 8q13.3 dup; 8q21.3 dup; 8q24.21 dup; 8q24.3 dup
29	*JAK2* V617F
30	5q22.2 del; 5q31.2 del; 5q33.1 del; 5q33.3 del
31	Normal genotype
32	8q13.3 dup; 8q21.3 dup; 8q24.21 dup; 8q24.3 dup
33	Normal genotype
34	Normal genotype
35	5q22.2 del; 5q31.2 del; 5q33.1 del; 5q33.3 del
36	Normal genotype
37	8p12 dup; 8q13.3 dup; 8q21.3 dup; 8q24.21 dup; 8q24.3 dup
38	Normal genotype
39	Normal genotype
40	Normal genotype
41	11q23.3 del
42	Normal genotype
43	Normal genotype
44	Normal genotype
45	Normal genotype
46	7q21.2 del; 7q22.1 del; 7q36.1 del
47	8p12 dup; 8q13.3 dup; 8q21.3 dup; 8q24.21 dup; 8q24.3 dup
48	5q22.2 del; 5q31.2 del; 5q33.1 del; 5q33.3 del
49	*JAK2* V617F
50	Normal genotype
51	Normal genotype
52	Normal genotype
53	Normal genotype
54	Normal genotype
55	Normal genotype
56	del 5q33.1
57	Normal genotype
58	Normal genotype
59	Normal genotype

60	5q31.1 del; 5q33.1 del; 5q33.3 del; 7p12.2 del; 7q21.2 del; 7q22.1 del; 7q22.2 del; 7q31.2 del; 7q36.1 del
61	Normal genotype
62	Yp11.31 dup
63	Normal genotype
64	*JAK2* V617F
65	Normal genotype
66	Normal genotype
67	*JAK2* V617F
68	Normal genotype

Supplementary Table S2: Detailed results of samples with no metaphases analyzed by MLPA.

Patient ID	CCA outcome (karyotyping)	MLPA Outcome	Genetic dinding (MLPA)
P-1	No metaphases detected (failed)	Successful	Normal genotype
P-11	No metaphases detected (failed)	Successful	Normal genotype
P-13	No metaphases detected (failed)	Successful	5q33.1 del, 5q31.2 del, 5q22.2 del
P-18	No metaphases detected (failed)	Successful	Normal genotype
P-24	No metaphases detected (failed)	Successful	Normal genotype
P-31	No metaphases detected (failed)	Successful	Normal genotype
P-34	No metaphases detected (failed)	Successful	Normal genotype
P-35	No metaphases detected (failed)	Successful	5q22.2 del, 5q31.2 del, 5q33.1 del, 5q33.3 del
P-41	No metaphases detected (failed)	Successful	11q23.3 del
P-45	No metaphases detected (failed)	Successful	Normal genotype
P-46	No metaphases detected (failed)	Successful	7q21.2 del, 7q22.1 del, 7q36.1 del

P-48	No metaphases detected (failed)	Successful	5q22.2 del, 5q31.2 del, 5q33.1 del, 5q33.3 del
P-50	No metaphases detected (failed)	Successful	Normal genotype
P-55	No metaphases detected (failed)	Successful	Normal genotype
P-56	No metaphases detected (failed)	Successful	del 5q33.1
P-57	No metaphases detected (failed)	Successful	Normal genotype
P-59	No metaphases detected (failed)	Successful	Normal genotype
P-60	No metaphases detected (failed)	Successful	5q31.1 del, 5q33.1 del, 5q33.3 del ,7p12.2 del, 7q21.2 del, 7q22.1 del, 7q22.2 del, 7q31.2 del, 7q36.1 del
P-65	No metaphases detected (failed)	Successful	Normal genotype
P-66	No metaphases detected (failed)	Successful	Normal genotype

Supplementary Table S3: Detailed results of patients lacking CCA due to non performance and analyzed by MLPA.

Patient ID	CCA outcome (karyotyping)	MLPA outcome	Genetic finding (MLPA)
P-4	Not conducted	Successful	*JAK2* V617F
P-22	Not conducted	Successful	Normal genotype
P-32	Not conducted	Successful	8q13.3 dup; 8q21.3 dup; 8q24.21 dup; 8q24.3 dup
P-49	Not conducted	Successful	*JAK2* V617F

Supplementary Table S4: Strengths and limitations of CCA, MLPA, and NGS for MDS diagnosis.

Factor	Conventional cytogenetics (CCA)	MLPA	Next-Generation

			Sequencing (NGS)
Primary target	Structural/numerical aberrations (whole chromosomes)	CNVs (deletions/duplications) at targeted loci	Point umtations, CNVs, *indels**, fusions
Sample requirement	Live, dividing cells (Bone Marrow/Blood)	Genomic DNA (bone marrow/blood)	High-quality genomic DNA ((bone marrow/blood)
Turnaround time (TAT)	7–14 days (requires cell culture)	1–2 days (fastest)	10–21 days (slowest)
Cost (per sample)	Medium (culture, reagents)	Low (most cost-effective)	High (equipment, consumables, bioinformatics)
Resolution (scope)	Low (min. ~5–10 Mb)	Medium (loci-specific)	High (single-nucleotide level)
Key limitation	High failure rate, low resolution	Cannot detect point mutations or balanced translocations	High initial cost, complex data analysis
Key advantage	Detects all large structural changes (unbiased)	High success rate, rapid, economical	Gold standard for modern prognostic risk stratification

Notes: Comparison based on: MLPA features and cost [11], [8], CCA limitations (culture failure) [27], and NGS role in modern risk stratification (IPSS-M) [29], [30], [31].
*indels: insertions and deletions.
